# Signal transducer and activator of transcription 3 activation is associated with bladder cancer cell growth and survival

**DOI:** 10.1186/1476-4598-7-78

**Published:** 2008-10-21

**Authors:** Chun-Liang Chen, Ling Cen, Jennifer Kohout, Brian Hutzen, Christina Chan, Fu-Chuan Hsieh, Abbey Loy, Victor Huang, Gong Cheng, Jiayuh Lin

**Affiliations:** 1 Center for Childhood Cancer, The Research Institute at Nationwide Children's Hospital, Columbus, OH 43205, USA; 2 Department of Pediatrics, The Ohio State University, Columbus, OH 43205, USA; 3 Biochmietry Program, The Ohio State University, Columbus, OH 43205, USA; 4 Integrated Biomedical Science Graduate Program, The Ohio State University, Columbus, OH 43205, USA; 5 Molecular, Cellular and Developmental Biology Program, The Ohio State University, Columbus, OH 43205, USA

## Abstract

**Background:**

Constitutive activation of signal transducer and activator of transcription 3 (Stat3) signaling pathway plays an important role in several human cancers. Activation of Stat3 is dependent on the phosphorylation at the tyrosine residue 705 by upstream kinases and subsequent nuclear translocation after dimerization. It remains unclear whether oncogenic Stat3 signaling pathway is involved in the oncogenesis of bladder cancer.

**Results:**

We found that elevated Stat3 phosphorylation in 19 of 100 (19%) bladder cancer tissues as well as bladder cancer cell lines, WH, UMUC-3 and 253J. To explore whether Stat3 activation is associated with cell growth and survival of bladder cancer, we targeted the Stat3 signaling pathway in bladder cancer cells using an adenovirus-mediated dominant-negative Stat3 (Y705F) and a small molecule compound, STA-21. Both prohibited cell growth and induction of apoptosis in these bladder cancer cell lines but not in normal bladder smooth muscle cell (BdSMC). The survival inhibition might be mediated through apoptotic caspase 3, 8 and 9 pathways. Moreover, down-regulation of anti-apoptotic genes (Bcl-2, Bcl-xL and survivin) and a cell cycle regulating gene (cyclin D1) was associated with the cell growth inhibition and apoptosis.

**Conclusion:**

These results indicated that activation of Stat3 is crucial for bladder cancer cell growth and survival. Therefore, interference of Stat3 signaling pathway emerges as a potential therapeutic approach for bladder cancer.

## Background

Several malignancies have been shown to result from constitutive activation of STATs, in particular Stat3 and 5 [1,2]. Stat3 is widely expressed in normal tissues and transiently activated and then inactivated by a group of signaling proteins, such as SH2-containing tyrosine phosphotases (SHP1 and SHP2), protein inhibitors of activated STATs (PIAS) and suppressor of cytokine signaling proteins/extracellular signaling regulated kinase (SOCS/ERK) cascades [3-5]. In a variety of human cancers, defects in these signaling pathways or persistent presence of up-stream activators would lead to constitutive activation of Stat3 and tumorgenesis [6,7]. Interference of constitutive Stat3 signaling pathway suppresses chemotherapy resistance, tumor growth and metastasis, induces cancer cell death and therefore shows great potential for cancer therapy [8,9].

Several lines of evidence suggest that constitutive activation of Stat3 might play a role in bladder malignancy. Bladder cancer is one of the common malignancies and molecular causes for its progress and development have been intensively investigated [10-12]. However, the detailed picture of oncogenic pathways for bladder cancer has just begun to be revealed [11]. Bladder cancer is induced by amplification of oncogenes [eg. fibroblast growth factor receptor 3 (FGFR3) and Ras gene] or by mutational defects in tumor suppressor genes (eg. PTCH & PTEN). These diverse genetic changes lead to oncogenic signalings via MAPK, PI-3 kinase, AKT and c-Myc pathways. Overactive FGFR3 and ERBB2 in bladder cancer presumably would activate Stat3 that is down-stream to these two receptor tyrosine kinases [10]. Another line of evidence is that overexpression of Stat3-regulated anti-apoptotic genes (Bcl-2, Bcl-xL and survivin) is found in bladder cancer. Overexpression of these genes renders bladder cancer progression, accelerated rates of recurrences, anti-apoptosis and chemotherapeutic resistance [13-18]. The role of activated Stat3 in bladder cancer remained speculative until the recent report showed that Stat3 activation correlated with malignant characteristics of T24 bladder cancer cells [19]. This implicates that activation of Stat3 may play a role in the development of bladder cancer.

We initiated a study to explore any further relation between activation of Stat3 and bladder malignancy. We found that 19 of 100 (19%) bladder cancer biopsy tissues had elevated expression of phosphorylated-Stat3 (p-Stat3) using an immunohistochemical staining with a p-Stat3 specific monoclonal antibody. In addition, elevated p-Stat3 expression was also found in bladder cancer cell lines, UMUC-3, 253J and WH. Thereafter, we targeted the activated Stat3 signal pathway using a dominant negative Stat3 Y705F (dnStat3) and a small molecule inhibitor, STA-21 [8,20]. Inhibition of Stat3 pathway suppressed cell growth of bladder cancer cells in vitro. DnStat3 and STA-21 also induced apoptosis as revealed by immunostaining of cleaved caspases 3, 8 and 9 in bladder cancer cells. Down regulation of anti-apoptotic genes (Bcl-2, Bcl-xL and survivin) and a cell-cycle regulating gene, cyclin D1, were correlated with dnStat3- and STA-21 induced apoptosis and cell growth inhibition. Taken together, Stat3 activation may play a pivotal role in bladder cancer cell growth and survival and serve as a novel therapeutic target for this type of cancer.

## Results

### p-Stat3 was elevated in bladder cancer tissues

**Figure 1:**
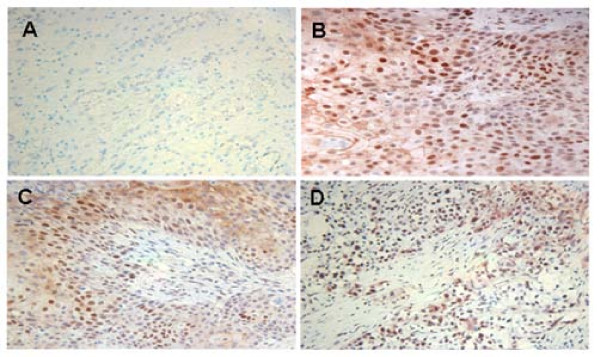
**p-Stat3 was elevated in bladder cancer tissues.** (A) normal tissue, (B) squamous cell carcinoma (Stage II), (C) urothelial carcinoma (stage III), (D) urothelial carcinoma (stage IV). Normal tissues appeared negative in p-Stat3 staining. The nuclei were counterstained with hematoxylin blue. Image magnification was 100×.

Tissue microarray immunohistochemistry indicated that Stat3 phosphorylation was elevated in bladder cancer tissues. Three representative bladder cancer tissues with p-Stat3 positive immunostaining (scale 2–3) are shown (Figure 1B–D), whereas normal bladder tissues were negative or very weak (scale 0–1) with immunostaining (Figure 1A). The elevated p-Stat3 in the bladder cancer tissues was scored and summarized according to immunostaining intensities. Two of the bladder cancer tissues were not included for immunostaining scoring because no staging information is available. Nineteen out of 100 bladder cancer tissues were positive for p-Stat3 immunostaining (scale 2–3).

The clinicopathological data for 102 bladder cancer tissues are classified in Table 1. The samples represented 76 male and 26 female patients, with the majority of patients (95.1%, 97 out of 102) between 41–90 years old. Forty-five tissues and 57 tissues were staged and graded, respectively. Forty of 45 staged tissues (89%) had no signs of regional lymph nodes nor distant organ metastases. According to histological features, the bladder cancer tissues were identified as urothelial carcinoma (85%), squamous cell carcinoma (3%), adenocarcinoma (2%) and mixed carcinomas (10%).

**Table 1:** Clinicopathological parameters of urinary bladder cancers used

Clinicopathological parameters		Numbers (%)
Gender	Male	76 (75.9)
	Female	26 (24.1)
		
Age (years)	20–40	4 (3.9)
	41–60	41 (40.2)
	61–90	56 (54.9)
	N/A^a^	1 (1.0)
	Mean	62
	Median	64
		
Stage (total 45)	0a^b^	6 (13.3)
	0is^c^	3 (6.7)
	I	16 (35.6)
	II	7 (15.6)
	III	8 (17.8)
	IV	3 (6.7)
	N/A	2 (4.4)
		
Grade (Total 57)	I	5 (8.8)
	II	17 (29.8)
	III	35 (61.4)
		
Regional lymph node and/or distant metastasis	N0M0^d^	40 (88.9)
(total 45)	N1M0^e^	1 (2.2)
	N0M1^f^	1 (2.2)
	N1M1^g^	1 (2.2)
	N/A	2 (4.4)
		
Histology (total 54)	Urothelial carcinoma	87 (85.3)
	Squamous cell carcinoma	3 (2.9)
	Adenocarcinoma	2 (2.0)
	Mixed carcinomas	10 (9.8)

^a ^N/A – Information are not available

^b ^0a – Cancer is noninvasive

^c ^0is – Noninvasive, flat carcinoma in situ

^d ^N0M0 – No regional lymph nodes neither distant organs metastasis

^e ^N1M0 – Metastasis in 1 to 3 regional lymph nodes

^f ^N0M1 – No regional lymph nodes but distant organs metastasis

^g ^N1M1 – Metastasis both in regional lymph nodes and distant organs

### p-Stat3 was also elevated in bladder cancer cell lines

**Figure 2:**
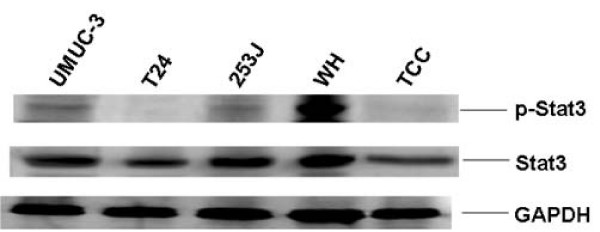
**Elevated p-Stat3 (Y705) was found in bladder cancer cell lines.** One hundred μg of cell lysates from bladder cancer cells were subject to western blot analysis using anti-p-Stat3 (Y705), -Stat3 and -GAPDH specific antibodies.

Western blot analysis showed that elevated p-Stat3 was also found in bladder cancer cell lines, UMUC-3, 253J and WH (Figure 2). Very low or no p-Stat3 was detected in the same amount of T24 and TCC cell lysates as revealed by the internal controls of GAPDH. All bladder cancer cell lines examined have very similar total Stat3 expression levels.

### rAd-mediated transduction of dnStat3 in bladder cancer cell lines

**Figure 3:**
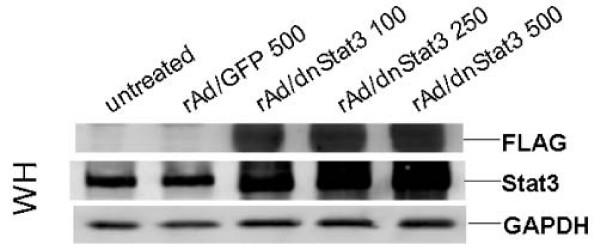
**Transduction of dnStat3 (Y705F) in bladder cancer cell lines using a recombinant adenoviral vector. **A representative bladder cancer cell line, WH, was transduced with designated mois of rAd/dnStat3 and rAd/eGFP. Twenty μg of cell proteins were fractionated on 12% PAGE gels and immunoblotted with specific antibodies against FLAG, Stat3 and GAPDH.

One way to investigate the functions of activation of Stat3 in bladder cancer is to interrupt Stat3 signaling pathway in bladder cancer cell lines with elevated p-Stat3. To that end, WH and UMUC-3 were transduced with rAd/dnStat3. The two cell lines were infected with rAd/dnStat3 (moi = 100, 250, and 500). The expression of dnStat3 in WH cells was observed and shown at 48 hours post infection (Figure 3). FLAG-tagged dnStat3 expressions in 10 μg of bladder cancer cell lysates were detected by western blots using an anti-FLAG antibody. Total Stat3 expressions correspondingly reflected the dose-dependent increase of dnStat3 expressions in these bladder cancer cells.

### Targeting Stat3 signaling pathway using dnStat3 and STA-21 induced cell growth and viability inhibition in bladder cancer cells

**Figure 4:**
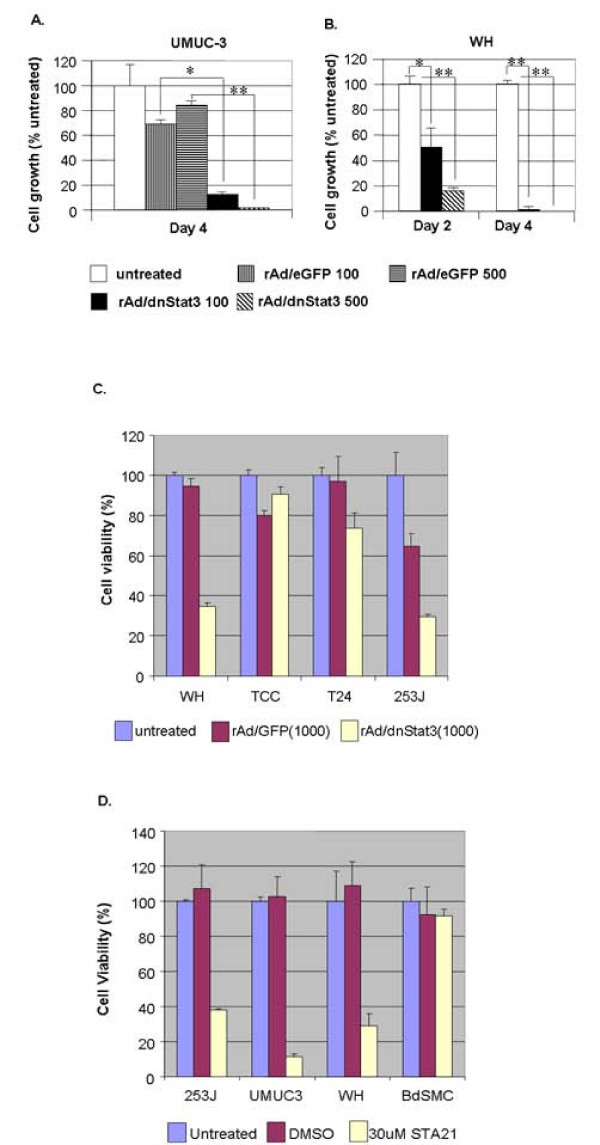
**Targeting Stat3 pathway inhibits cell growth and viability.** Transduction of dnStat3 suppressed cell growth of bladder cancer cell lines, (A) UMUC-3, at day 4 post infection and (B) WH, at day 2 and 4 post infection. Growth of bladder cancer cells was strongly suppressed by the expression of dnStat3. Cells were transduced with either rAd/dnStat3 or rAd/eGFP (moi = 100 or 500) while untransduced cells served as negative controls. Cells in five random individual microscopic fields (100×) were scored at day 2 or day 4 post-infection. (C) MTT assay also shows that transduction of dnStat3 limits cell growth of bladder cancer cell lines (WH and 253J) with higher content of p-Stat3 but not that of those cells with less p-Stat3 at 4 day post-infection. Cell growth was shown in cell density over control cell density (%). (D) Cell viability of cancer cells was greatly reduced after treatment of STA-21, a Stat3 dimerization inhibitor. Cells were treated with DMSO or 30 μM STA-21 for 4 days. STA-21 had very limited effects on BdSMC cell viability. Cell viability was determined using a MTT assay. Averages and standard deviations for data points were derived from triplicate experiments. * (P < 0.05) and ** (P < 0.005) indicate paired t-test statistic significance.

Bladder cancer cell growth was significantly suppressed in the presence of dnStat3 and STA-21. UMUC-3 and WH cells were transduced with either rAd/dnStat3 or rAd/eGFP (moi = 100 and 500). Cell growth is presented in cell densities normalized to untransduced controls at day 2 and day 4 post-infection (Figure 4A and 4B). The growth rates of untransduced cells were set at 100%. There was 20–30% reduction in UMUC-3 cell growth at day 4 post-infection with rAd/eGFP; more notably, the UMUC-3 cell line transduced with rAd/dnStat3 (moi = 100 and 500) was reduced down to 11.9 ± 0.3% (P < 0.05) and 1.3 ± 0.3% (P < 0.005) of cell growth at day 4 compared to untransduced controls (Figure 4A). The discrepancies between cell growth of bladder cancer cells transduced with rAd/eGFP and rAd/dnStat3 apparently reflects dnStat3-specific inhibitory effects. The dnStat3-mediated inhibition of cell growth was even more dramatic as observed in WH cells (Figure 4B). WH cells transduced with rAd/dnStat3 (moi = 100 and 500) had only 50.2 ± 13.5% (P < 0.05) and 16.1 ± 2.2% (P < 0.005) of cell growth compared to untransduced controls at day 2 post-infection; evenmore, at day 4 post-infection, cell growth of rAd/dnSTat3-transduced WH cells was decreased to only 1.1% and 0.5% of untransduced controls. After 4 day of infection with a higher dose of rAd/Stat3 (moi = 1000), a cell viability assay (MTT) also revealed that WH and UMUC with higher content of p-Stat3 only maintained 34.8 ± 1.4% and 51.9 ± 8.5% cell viability of untreated controls (Figure 4C). On the contrary, TCC and T24 with much lower or undetectable p-Stat3 contents had much higher cell viability with 90.5 ± 3.6% and 73.6 ± 7.5% of untreated controls under the same experimental conditions. The overall cell viability of cells treated with rAd/dnStat3 was decreased along the time, while that of cells untransduced or treated with rAd/eGFP was increased (data not shown).

As MTT assay showed, similar inhibition on cell growth and viability was observed in bladder cancer cells treated with 30 μM STA-21 (Figure 4C). Viability of bladder cancer cells and BdSMC treated with DMSO was about the same as untreated controls. However, exposure to STA-21 greatly reduced cell viability of 253J, UMUC3 and WH (38.1 ± 0.74%, 11.4 ± 1.5%, and 29.0 ± 6.7%). Interestingly, STA-21 had very minimal effects on BdSMC cell viability (91.3 ± 4.4%). This indicated that STA-21 inhibition is specific to bladder cancer cells that have constitutive activation of Stat3. In addition, the decreased overall viability of cells treated with STA-21 was consistent with that observed in cells treated with rAd/dnStat3. These together suggested that bladder cancer cell death associated with inhibition of Stat3 pathway might have occurred.

### Inhibition of Stat3 pathway induces activation of apoptotic caspase pathways

**Figure 5:**
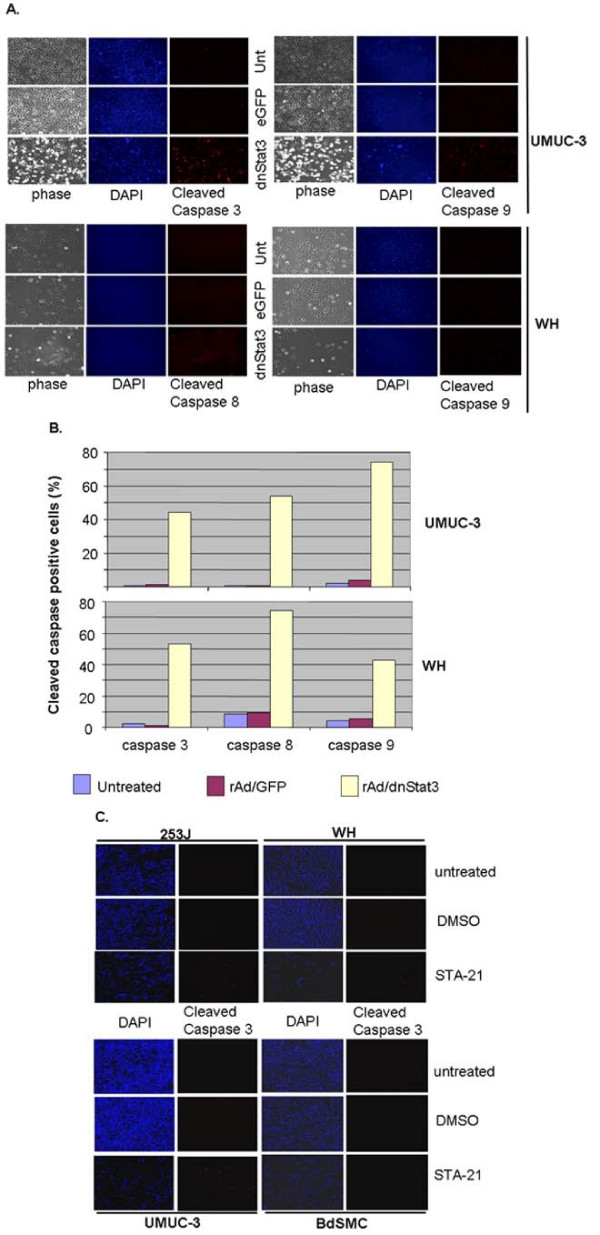
**Inhibition of Stat3 pathway induces apoptosis through caspase 3, 8 and 9 pathways in bladder cancer cells but not in bladder smooth muscle cells.** (A) Cleaved caspase 3, 8 and 9 staining in dnStat3-transduced UMUC-3 and WH at 48 h post-infection and 31 h post-infection, respectively. Cells were transduced, fixed and then immunostained with anti-cleaved caspases 3, 8 and 9 antibodies. Cleaved caspases 3, 8 and 9 immunoreactivies were observed in cells transduced with rAd/dnStat3 but not in the cells transduced with rAd/eGFP or negative control. Cleaved caspase 3, 8, 9: anti-cleaved-caspases 3, 8, & 9 antibody immuno-fluorescent staining; Unt: untreated; DAPI: nuclear staining with DAPI; phase:phase-contrast. Magnification of images was 100×. (B) 45–75% of UMUC-3 and WH cells transduced with rAd/dnStat3 were cleaved caspase 3, 8, and 9 positive. (C) STA-21 also induced apoptosis (cleaved caspase 3 staining) in 253J, WH and UMUC-3 cells but not in BdSMC cells.

Transduction of dnStat3 in bladder cancer cells induced activation of apoptotic caspases 3, 8, and 9 in those cells transduced with rAd/dnStat3. UMUC-3 and WH were fixed at day 2 and day 1, respectively, post-transduction of rAd/eGFP or rAd/dnStat3 and then subject to immuno-fluorescent staining using antibodies that recognize cleaved caspases 3, 8 and 9 for apoptosis evaluations. Only rare sporadic cells were found stained by anti-cleaved caspases 3, 8, and 9 antibodies in negative UMUC-3 and WH controls (untransduced or transduced with rAd/eGFP) (Figure 5A &5B). However, many dnStat3-transduced cells were positive in anti-cleaved caspases 3 (44.2 and 53%), 8 (53.5 and 74.4%), and 9 (74.2 and 42.4%) immunostaining (Figure 5B).

Targeting Stat3 pathway using STA-21 also led to increased cleavage of caspase 3 after 72 hours of treatment in 253J, UMUC-3 and WH (Figure 5C and data not shown). About 6.4–13% cells were cleaved caspase 3 positive in these three STA-21-treated bladder cancer cell lines, compared to less than 0.5% in untreated or DMSO-treated cells. STAT-21 treatment did not seem to induce caspase 3 cleavage in BdSMC cells with less than 0.1% appearing positive with anti cleaved caspase 3 immunoreactivity. The increased apoptotic caspase activation implicated that apoptosis could be one of mechanisms underlying the decreased cell viability in bladder cancer cells in that Stat3 pathway was compromised by treatment of rAd/Stat3 or STA-21.

### dnStat3 down-regulated anti-apoptotic genes and cyclin D1 in bladder cancer cells

**Figure 6:**
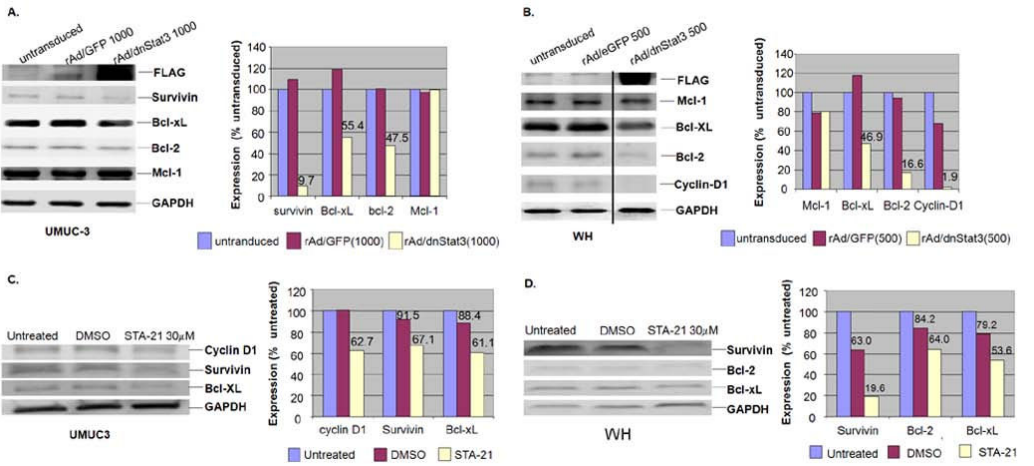
**Inhibition of Stat3 pathway by dnStat3 down-regulates survival genes and cyclin-D1 gene.** The protein expression levels are shown in (A) UMUC-3 at 27 hours and (B) WH at 48 hours post-infection with rAd/dnStat3 or the control vector, rAd/eGFP as well as in (C) UMUC-3 and (D) WH that are treated with designated concentrations of STA-21 for 4 days. Protein expressions of survival genes (Bcl-xL, Bcl-2, Mcl-1 and survivin) and cyclin-D1 were subject to densitometric quantification and shown in percents of the untreated controls after being normalized to the GAPDH expression. A representative experiment of triplicates is shown.

We then would like to explore possible mechanisms for dnStat3-induced cell growth inhibition and activation of apoptotic caspases in bladder cancer cells. It is likely that inhibition of Stat3 pathway by dnStat3 or STA-21 down regulated Bcl-2, Bcl-xL and survivin as well as cell cycle regulating gene, cyclin D1. Reduced expressions of these genes may contribute to bladder cancer cell growth inhibition and apoptotic caspase activation. UMUC-3 and WH cells were transduced with either rAd/eGFP or rAd/dnStat3 (moi = 1000 and 500) for 27 hours and 48 hours, respectively. Expression of survivin, Mcl-1, Bcl-2, Bcl-xL and cyclin D1 proteins in these cells were evaluated using western blot analysis and densitometric quantification. In UMUC-3 cells, Bcl-2 (47% untreated control), Bcl-xL (55.4%) and survivin (9.7%) were down regulated by the transduction of dnStat3 as compared to expressions in untransduced cells or cells transduced with rAd/eGFP (Figure 6A), whereas Mcl-1 (100%) remained intact. Expression of the three genes in rAd/eGFP-transduced cells (moi = 1000) was only slightly changed as compared to the untransduced cells. survivin(67.1% untreated control), Bcl-xL (61.1%) and cyclin -D1 (62.7%) expression was detected to be decreased when the same cell line treated with 30 μM STA-21 for 4 days (Figure 6C).

We also examined the expression of anti-apoptotic genes and cyclin D1 in WH bladder cancer cells when Stat3 pathway was targeted by rAd/dnStat3 or STA-21. Mcl-1 (80% untreated control) was apparently not affected as compared with the control treated with rAd/eGFP but Bcl-2 (16.6%) and Bcl-xL (46.9%) protein expressions were down regulated by the transduction of dnStat3 (Figure 6B). In addition, cyclin D1 expression (1.9% untreated control) was almost completely inhibited by the interference of Stat3 signaling pathway. Expression reduction in two anti-apoptosis genes (Bcl-2 and Bcl-xL) and cyclin D1 were consistent with apoptosis and cell growth inhibition in WH cells. Four days of treatment of STA-21 showed similar but less reduction in survivin (19.6% untreated control), Bcl-2 (64%) and Bcl-xL (79.2%) expressions.

## Discussion

Constitutive activation of Stat3 signaling pathway is frequently detected in several types of human cancers. This report was to explore the correlation between bladder cancer and Stat3 status in bladder cancer tissues and cell lines. We found that elevated p-Stat3 expression is found in both bladder cancer tissues and cell lines. Among 100 primary bladder cancer biopsy tissues, 19% appears positive in p-Stat3 immunostaining in nuclei, cytoplasm or both compartments. Majorities of bladder cancer tissues examined are negative for p-Stat3 and may result from other causes for this kind of cancer [10]. Elevated p-Stat3 expression is also found in bladder cancer cells, UMUC-3, WH and T24. These suggest that elevated p-Stat3 might contribute to some of bladder malignancy. Phosphorylation at tyrosine 705 is required for the activation of Stat3. Elevated Stat3 phosphorylation in these bladder tissues and cell lines might result from abnormal overactive upstream oncogenic FGFR or ERBB2 in these cancer tissues [10,21]. A recent study shows that overactive Stat3 serves as the signal mediator between EGF and MMP-1 for bladder cancer cell migration, invasion and tumor formation [19]. Alternative explanation is the down regulation of counter balancing signal transduction pathways, such as SH2-containing tyrosine phosphotase (SHP1 and 2), protein inhibitors of activated Stats (PIAS), and suppressors of cytokine signaling (SOCS), could also contribute to higher Stat3 phosphorylation in these bladder cancer tissues or cell lines [5]. These need further verifications using tissue microarray immunohistochemistry or quantitative PCR.

Our data suggest that bladder cancer cells might utilize Stat3 signaling pathway for cell growth and survival. Interruption of Stat3 pathway using a dnStat3 or STA-21 affects bladder cancer cell growth and induces the activation of apoptotic caspases. DnStat3 may inhibit phosphorylation and dimerization of endogenous Stat3 [22-24] and down regulates a group of survival and proliferation genes [25-27]. STA-21 was discovered from a virtual drug screen and showed efficacy in blocking Stat3 dimerization and translocation into nuclear compartments [8]. Our data, consistent with previous studies, has delineated part of the relationship between elevated p-Stat3 expression and bladder cancer [19], although mechanisms for cell growth inhibition and cell death by dnStat3 in bladder cancer cell UMUC-3 and WH remain largely unclear. Reduction of cyclin-D1 expression in WH and UMUC-3 cells might be part of the causes for cell growth inhibition. This is consistent with previous study that targeting Stat3 signaling with dnStat3 suppresses cell-cycle-related genes, including cyclin-D1, in ALK-positive anaplastic large cell lymphoma [28].

Interruption of Stat3 pathway by dnStat3 and STA-21 leads to activation of caspase 3 signaling in bladder cancer cells. Apparently, dnStat3-induced cleavage of caspase 3 is also mediated through caspases 8 and 9 pathways. Caspases 8 and 9 are key initiator caspases for two largely independent apoptotic pathways mediated by death receptors and stresses [29-32]. Cleaved caspase 8 suggests an autocrine signal(s) following dnStat3 transduction in bladder cancer cells. Fas, TRAIL receptors and their ligands are usually suppressed in several cancers to prevent apoptosis [33,34]. Stat3 has been shown to directly down regulate Fas, TRAIL, and TGF-α [35-37]. To Target Stat3 signaling pathway using Stat3β upregulates TRAIL and a secretory apoptotic signal(s) in B16 tumor cells. What death receptor(s) is involved in dnStat3-induced apoptosis in bladder cancer cells acquires further investigations.

Activation of caspase 9 pathway in bladder cancer cells is very likely triggered by down regulation of Bcl-2 family genes and inhibitors of apoptotic proteins (IAP). We observed that two Bcl-2 family genes (Bcl-2 and Bcl-xL) and an IAP gene (survivin) are negatively affected at protein level by dnStat3 and STA-21. Overexpression of Bcl-2, Bcl-xL, and survivin in several cancers overcomes severe tumor environments and facilitates cancer progression, chemotherapeutic resistance and higher rate of recurrence [15,17,18,38,39]. Down regulation of these genes likely contributes to the dnStat3- and STA-21-induced activation of apoptotic caspases in bladder cancer cells. DnStat3 also inhibits Stat3 signaling in ALK-positive anaplastic large cell lymphoma by suppression of several Bcl-2 family genes [28]. Activated Stat3 promoting cancer survival and proliferation has been demonstrated in several cancers [8,40-48]. To suppress Stat3 signaling pathway using anti-sense RNA, siRNA, small molecules, decoy-oligos and dnStat3 results in cancer cell growth inhibition and apoptosis. It appears that targeting dnStat3 signaling pathway could be an effective therapeutic approach for bladder cancer expressing constitutive activation of Stat3.

## Conclusion

Our data show that Stat3 phosphorylation is elevated and may play a pivotal role in cell growth and survival of bladder cancer. Cell growth inhibition and apoptosis can be induced in bladder cancer cell lines using either a dnStat3 or a small molecule inhibitor, STA-21 to interfere with the Stat3 signaling pathway. The Stat3 signaling pathway appears as a potential target for bladder cancer therapy.

## Methods

### Cell culture

Bladder cancer cell lines were purchased from American Type Culture Collection (ATCC). Cell lines were maintained in 1× DMEM supplemented with 10% fetal bovine serum and 100 U/ml penicilin/streptomycin/amphotericin B (Mediatech, Herndon, VA) at 37°C, aired with 5% CO_2_. Bladder smooth muscle cells (BdSMC) were purchased from Cambrex Bio Science and maintained in SmGM^®^-2-Smooth muscle medium (Cambrex, Chicago, IL) supplemented with 5% FBS.

### Bladder cancer tissue microarray immunohistochemistry

Stat3 phosphorylation status in bladder cancer tissues were examined using immunohistochemistry with a p-Stat3 (Y705)-specific monoclonal antibody (Cell Signaling Tech., Danvers, MA). We stained bladder cancer tissue samples (n = 102) on tissue microarray slides from two different providers (US Biomax, Inc., Rockville, MD and ISU ABXIS Co., Seoul, Korea). The immunohistochemistry and scoring of p-Stat3 expression were described previously [49]. Most of the p-Stat3 positive cancer tissues showed staining in greater than 50% of each sample.

### Western blots

Western blots were carried out according to protocols described previously [49]. 10 to 100 μg of cellular proteins were resolved on 10% or 14% SDS-PAGE gels before transfer, immunoblotting, and visualization of specific protein bands. Antibodies were purchased separately and used to recognize FLAG (Sigma, St. Louis, MO) GAPDH (Chemicon International, Temecula, CA). Stat3, p-Stat3 (Y705) (Cell Signaling Tech., Danvers, MA), Bcl-2, Bcl-x_L _(Biosciences, Inc. Franklin Lakes, NJ,), Mcl-1, cyclin D1 (Lab Vision Corp., Fremont, CA) and survivin (UpState, Charlotteville, VG)

For expression comparison, each protein expression was presented in a percentage of its corresponding untreated control after densitometric quantification and normalization to the GAPDH expression. A representative one from duplicated experiments was presented.

### Transduction of dnStat3 in bladder cancer cell lines

The construction of recombinant Adenovirus/CMV-dnStat3 Y705F (rAd/dnStat3) is described previously [23]. DnStat3 was generated from Stat3 by changing the tyrosine at position 705 into phenylalanine. The dnStat3 protein product is tagged with a 6-repeat FLAG sequence for detection and cannot be activated through tyrosine phosphorylation. About 2 × 10^5 ^WH and UMUC-3 cells were transduced with rAd/dnStat3 or rAd/CMV-eGFP (rAd/eGFP) (Applied Viromics, Fremont, CA) at variant multiplicities of infection (moi) based on TCID50 assay using 293T cells. For cell growth experiments, cell numbers were enumerated at day 2 or 4 post-infection. Cell counts in 5 random fields of view (magnification 100×) were obtained for each treatment and control. Cell growth rates were presented in percentages of cell density of untreated controls and averaged from triplicate experiments.

### Treatment of STA-21 and Cell viability assay

Approximately 5000 cells were grown in 100 μl 10% FBS-supplemented DMEM medium in 96-well flat-bottomed plates overnight. Cells were exposed to STA-21 (30 μM) that was dissolved in dimethyl sulfoxide (DMSO) before added to the medium. Cell viability was analyzed by the MTT [3-(4, 5-dimethyl-2-thiazolyl)-2, 5-diphenyl-2H-tetrazolium bromide] (Sigma) assay in three replicates. At the time of assay end-point, cells were treated with MTT (1 mg/ml) for 3–4 hours. Colormetric quntitation was determined by an EL808 Ultra Microplate Reader (Bio-Tek Intruments, Inc) after formazan was dissolved in 25% N, N-dimethylformamide and 10% SDS in a light-proof condition overnight.

### Caspases 3, 8, and 9 immuno-fluorescent staining

Approximately 1 or 2 × 10^5 ^cells (UMUC-3, WH, 253J, and BdSMC) were seeded on sterile coverslips in a 6-well plate overnight, either transduced by either rAd/eGFP or rAd/dnStat3 (moi = 500) for 48 or 28 hours respectively, for UMUC-3 and WH cells. For the small molecule inhibition, cells were treated with 30 μM STA-21 for 72 hours. Cleaved caspase immunostaining and documentation were described previously [50]. The primary rabbit antibodies were diluted with 1:100, 1:50, and 1:100 dilutions, respectively, for detecting cleaved-caspase-3 (Asp175), cleaved-caspase-8 (Asp374), or cleaved-caspase-9 (Asp330) (Cell Signaling Tech)

## Abbreviations

BdSMC: bladder smooth muscle cell; DMSO: dimethyl sulfoxide; MTT: [3-(4, 5-dimethyl-2-thiazolyl)-2, 5-diphenyl-2H-tetrazolium bromide]; PIAS: protein inhibitors of activated STATs; SHP1/2: SH2-containing tyrosine phosphotases; Stat3: signal transducer and activator of transcription 3.

## Competing interests

The authors declare that they have no competing interests.

## Authors' contributions

CLC participated in experiment designs, coordinated the experiments, contributed to the analysis and interpretation of data, and drafted the manuscript. LC and JK carried out the Adeno-viral dnStat3 and STA-21 experiments. BH participated in cell growth inhibition experiments. AL and VH carried out western blot analysis. CC, FCH and GC, carried out immunohistochemical staining of tissue microarray slides. JL conceived the ideas, coordinated the experiments and supervised on the data analyses, interpretation and the manuscript draft. All authors read and approved the final manuscript.
